# Identification of serum exosomal metabolomic and proteomic profiles for remote ischemic preconditioning

**DOI:** 10.1186/s12967-023-04070-1

**Published:** 2023-04-03

**Authors:** Yang Du, Rui Qiu, Lei Chen, Yuewen Chen, Zhifeng Zhong, Peng Li, Fangcheng Fan, Yong Cheng

**Affiliations:** 1 Key Laboratory of Ethnomedicine of Ministry of Education, Center on Translational Neuroscience, School of Pharmacy, Minzu University of China, Beijing, China; 2 Institute of National Security, Minzu University of China, Beijing, China; 3 Chinese Academy of Sciences Key Laboratory of Brain Connectome and Manipulation, The Brain Cognition and Brain Disease Institute, Shenzhen Key Laboratory of Translational Research for Brain Diseases, Shenzhen Institute of Advanced Technology, Chinese Academy of Sciences, Shenzhen–Hong Kong Institute of Brain Science–Shenzhen Fundamental Research Institutions, Shenzhen, 518055 China; 4 Shenzhen College of Advanced Technology, University of Chinese Academy of Sciences, Beijing, 100049 China; 5 Department of High Altitude Operational Medicine, College of High Altitude Military Medicine, Army Medical University, (Third Military Medical University), Chongqing, China; 6 College of Life and Environmental Sciences, Minzu University of China, Beijing, China

**Keywords:** Remote ischemic preconditioning, Metabolomic, Proteomic, Exosome

## Abstract

**Background:**

Remote ischemic preconditioning (RIPC) refers to a brief episode of exposure to potential adverse stimulation and prevents injury during subsequent exposure. RIPC has been shown to increase tolerance to ischemic injury and improve cerebral perfusion status. Exosomes have a variety of activities, such as remodeling the extracellular matrix and transmitting signals to other cells. This study aimed to investigate the potential molecular mechanism of RIPC-mediated neuroprotection.

**Methods:**

Sixty adult male military personnel participants were divided into the control group (n = 30) and the RIPC group (n = 30). We analyzed the differential metabolites and proteins in the serum exosomes of RIPC participants and control subjects.

**Results:**

Eighty-seven differentially expressed serum exosomal metabolites were found between the RIPC and control groups, which were enriched in pathways related to tyrosine metabolism, sphingolipid metabolism, serotonergic synapses, and multiple neurodegeneration diseases. In addition, there were 75 differentially expressed exosomal proteins between RIPC participants and controls, which involved the regulation of insulin-like growth factor (IGF) transport, neutrophil degranulation, vesicle-mediated transport, etc. Furthermore, we found differentially expressed theobromine, cyclo gly-pro, hemopexin (HPX), and apolipoprotein A1 (ApoA1), which are associated with neuroprotective benefits in ischemia/reperfusion injury. In addition, five potential metabolite biomarkers, including ethyl salicylate, ethionamide, piperic acid, 2, 6-di-tert-butyl-4-hydroxymethylphenol and zerumbone, that separated RIPC from control individuals were identified.

**Conclusion:**

Our data suggest that serum exosomal metabolites are promising biomarkers for RIPC, and our results provide a rich dataset and framework for future analyses of cerebral ischemia‒reperfusion injury under ischemia/reperfusion conditions.

**Supplementary Information:**

The online version contains supplementary material available at 10.1186/s12967-023-04070-1.

## Introduction

Remote ischemic preconditioning (RIPC) is a promising method for the protection of distant target organs when tissues or organs are exposed to intermittent ischemia/reperfusion conditions [1]. The organs achieve adaptive transient resistance to lethal ischemic injury through short-duration sublethal/mild ischemic injury preconditioning [2]. Recently, various types of RIPC have been performed experimentally to protect the brain, heart, kidney, and other organs [3].

Cerebrovascular accident or stroke is the second leading cause of death and a major cause of long-term disability worldwide, with an annual mortality rate of approximately 5.5 million. It is the main cause of global disability, with 50% of survivors suffering from chronic disability [4, 5]. Research has indicated that the incidence of stroke is increasing, and one-quarter of people experience stroke in their lifetime worldwide [6]. Stroke is classified as ischemic or hemorrhagic. It has been suggested that ischemic stroke is the most common form of stroke in the world [7]. Ischemic stroke is caused by transient or permanent occlusion of cerebral vessels, resulting in cellular damage in the brain and neurologic disability [8, 9]. Neurologic disability, including difficulties with memory, impaired reflexes, cognitive impairment, and aphasia, reduces quality of life [10]. Therefore, brain protection is a key objective in a variety of relevant clinical settings. Several pieces of preclinical evidence support the effectiveness of RIPC in inducing neuroprotection against cerebral ischemia‒reperfusion injury [11]. RIPC is now commonly carried out on limbs with blood pressure cuffs that inflate to prevent blood perfusion [12]. In addition, it has been indicated that preconditioning could increase tolerance to ischemic injury and improve cerebral perfusion status [13, 14]. Previous studies have reported the neuroprotective benefits of RIPC on ischemia/reperfusion injury [15]. Moreover, RIPC has been indicated to reduce injury in an experimental model of ischemic stroke and reduce injury and neurological sequela in humans after cardiac surgery [16, 17]. RIPC can effectively induce tolerance to cerebral ischemia, thereby reducing ischemic injury and improving the prognosis of patients. However, the underlying mechanisms of this process are not fully understood.

Exosomes play essential roles in cell-to-cell communication and have a variety of activities, such as remodeling the extracellular matrix and transmitting signals to other cells [18]. This intercellular vesicular transport pathway plays a critical role in many aspects of human health and disease, including development, tissue homeostasis, immunity, and neurodegenerative diseases [19]. Recently, exosomes have gained more attention in the regulation of diseases based on metabolome and proteome characterization [20]. Metabolomics and proteomics have been widely used to study complex systems [21, 22]. The metabolite spectrum that is generated is considered to be an effective indicator of biological physiology, and metabolite analysis assesses the interaction among a variety of proteins, genes, and the environment [23]. In this study, we performed ultraperformance liquid chromatography-tandem mass spectrometry (UPLC‒MS/MS) and liquid chromatography-tandem mass spectrometry (LC‒MS/MS) to analyze the serum exosome metabolomic and proteomic profiles associated with RIPC.

## Materials and methods

### Participants

Sixty adult male military personnel participated in the study. All participants were physically healthy and completed the medical questionnaire before the test. Exclusion criteria: (1) acute or chronic diseases, including generalized anxiety, depression, cardiovascular disease, respiratory system disease, movement, and metabolic disease; (2) habit of drinking or smoking; (3) having taken medicines in 3 months; (4) high altitude (> 2500 m) exposure; (5) participation in clinical trials within 3 months. Participants were divided into the control group (n = 30) and the RIPC group (n = 30). The main characteristics of all participants are shown in Table 1. All participants provided written informed consent. The study protocol was approved by the ethics review board of the Minzu University of China and was conducted according to the guidelines of the Declaration of Helsinki.

**Table 1:** Demographic characteristics of the subjects

Feature	Control group	RIPC group
n	30	30
Age	22.67 ± 1.83	22.23 ± 1.45
Weight	67.87 ± 7.76	65.77 ± 8.48
Height	173.37 ± 5.48	172.1 ± 6.50

*RIPC* Remote ischemic preconditioning

Values are expressed as the mean ± standard deviation

### Protocol for RIPC paradigm

A blood pressure cuff was placed around the left and right upper arms of participants in normal oxygen conditions. This paradigm involved inflation at a pressure of 80 mmHg for 5 min to block blood flow and then deflation for 5 min. The protocol was repeated for four cycles, which took 40 min (5 min of arterial occlusion + 5 min of arterial nonocclusion). The control group was treated identically without RIPC treatment. This treatment was performed daily for 10 days at sea level, and peripheral venous blood samples were obtained on the tenth day.

### Exosome isolation and validation

Serum exosome isolation was performed using size-exclusion chromatography (qEV column, 70 nm; Izon, Oxford, UK). Exosome validation was performed using negative-staining electron microscopy, nanoparticle tracking analysis (Additional file 1), and Western blot methods, which were described previously [24]. ZetaView (version: 8.04.02 SP2) analysis showed a particle peak at approximately 100 nm, the duration of acquisition was 5 min, and the concentration of each sample was 2E + 12 particles/mL in 100 µl PBS for metabolite and proteome measurements.

### Metabolite measurements

Widely targeted metabolomic analysis of serum exosome samples from participants was performed using the UPLC‒MS/MS method as described previously [25]. Briefly, MetaWare (a public database of metabolite information and metabolomics data management environment) was used for qualitative analysis of first- and second-order mass spectrometry. The quantification of metabolites was carried out by multiple reaction monitoring and triple quadrupole mass spectrometry.

### Proteome measurements

Forty microliters of serum exosome samples was transferred into 100 μl of acetonitrile. After mixing, the samples were placed into liquid nitrogen for quick freezing, and the samples were concentrated by centrifugation. Then, 20 µl of 8 M urea (dissolved in 50 mM ABC solution) and 2 mM Tris (2-chloroethyl) phosphate (TCEP) were added to each sample sequentially, followed by heating at 55 °C for 30 min. Then, 14 mM indole acetic acid (IAA) was added to the samples and reacted in the dark at room temperature for 40 min. Then, 10 mM dithiothreitol (DTT) was added to the samples to stop the reaction (placed in a refrigerator at -20 °C). The concentration and purity of the proteins were quantitated by a SpectraMax QuickDrop (Molecular Devices, State of California, USA). The proteome measurements were performed according to the LC‒MS/MS method, which was described previously [26]. Briefly, Skyline with the UniProt database was used for the quantification of the targeted proteome. It should be noted that we mixed six individual samples into one sample for proteome measurements, and we adjusted the concentration of each sample to 2E + 12 particles/mL in 100 µl PBS for metabolite and proteome measurements.

### Differential expression analysis

Based on the detected proteins and metabolites, an orthogonal partial least squares-discriminant analysis (OPLS-DA) model was generated to assess differentially expressed (DE) metabolites and proteins, and variable importance in projection (VIP) was extracted from this model [27]. Differential metabolites were defined as those with VIP > 1.5 [28] and *P* < 0.05 by a Mann–-Whitney U test.

### Bioinformatics analysis

To understand the biological functions of the DE metabolites and proteins, metabolites were annotated using the Kyoto Encyclopedia of Genes and Genomes (KEGG) database (the major public database on metabolic pathways) and then mapped to the KEGG pathway database by MetaboAnalyst software [29]; Metascape pathway enrichment analysis was used for DE proteins [30]. Significant enrichments were defined as pathways with *P* < 0.05.

The Weighted Gene Coexpression Network Analysis (WGCNA) R software package was used for the coexpression network analysis. Pearson's correlation was performed to assess correlations between metabolite levels. Significant module-trait results were defined as Benjamini‒Hochberg (BH)-corrected *P* < 0.05. To further explore the biological function, metabolite-protein interaction pathway analysis was performed by MetaboAnalyst software [29].

### Statistical analysis

Unsupervised principal component analysis (PCA) was performed by the statistical function prcomp in R [25]. The potential of blood exosomal metabolites to discriminate between participants with RIPC and controls was evaluated using a receiver operating characteristic (ROC) curve generated by MetaboAnalyst software [29].

## Results

### Differential expression of serum exosomal metabolites

**Fig. 1:**
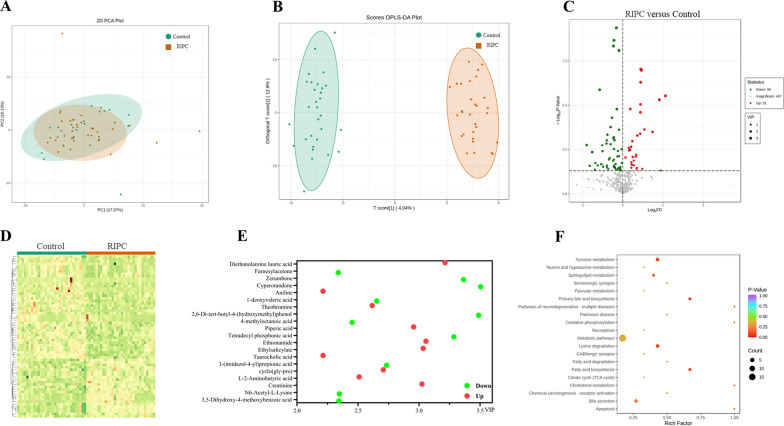
Bioinformatic screening for differential metabolites in serum exosomes. **A** Principal component analysis (PCA) and **B** orthogonal partial least squares-discriminant analysis (OPLS-DA) model plot in the participant set; **C** Volcano plot displaying differences in metabolite levels between RIPC participants and controls; **D** Heatmaps of the cluster analysis data; Metascape enrichment network analysis for **E** top 20 enriched metabolites and **F** top 20 enriched pathways. The node size is proportional to enrichment pathways; the bubble color represents the *P* value, and the color change from red‒green-purple represents a small to large transition of the *P* value

We performed UPLC‒MS/MS to analyze metabolomic profiles in serum exosomes in controls and RIPC participants. PCA plot scores showed distinct metabolite profiles for controls and preconditioning participants (Fig. 1A). An OPLS-DA model was used to identify differential exosomal metabolites between the two groups (Fig. 1B). Of these 87 metabolites, 56 had increased levels and 31 had decreased levels in the RIPC participants compared to the controls (Figs. 1C, D, Additional file 2). We used Metascape enrichment analysis to assess the differential metabolites. Figure 1E shows the top 20 enriched metabolites, including diethanolamine lauric acid, famesylacetone, zerumbone, cyperotundone, and aniline phenylacetone. Bioinformatics analyses showed the top 20 enrichment pathways, including tyrosine metabolism, taurine and hypotaurine metabolism, sphingolipid metabolism, serotonergic synapse, pathways of multiple neurodegeneration diseases, Parkinson's disease, and GABAergic synapse. (Fig. 1F).

### Perturbation of serum exosomal metabolite coexpression modules

**Fig. 2:**
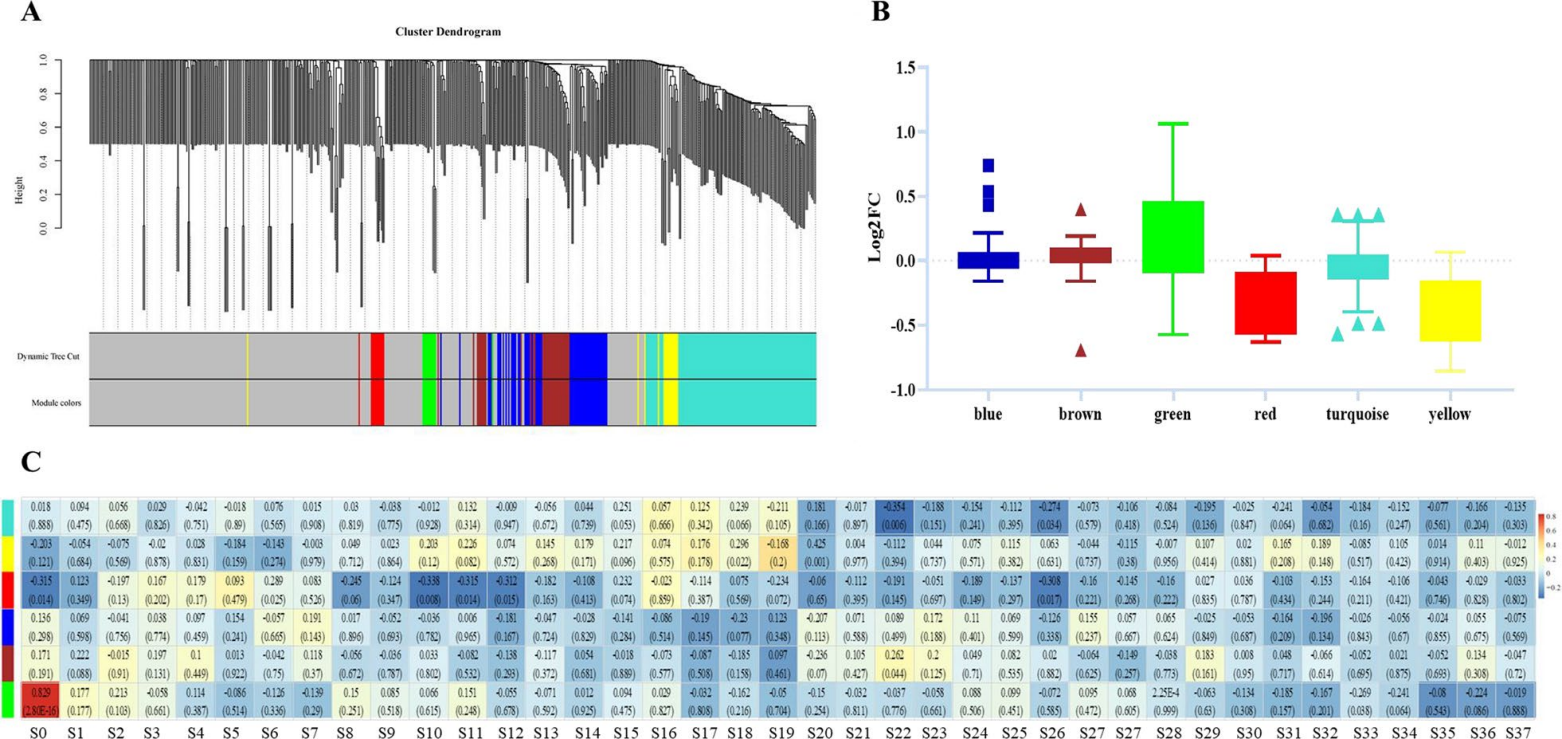
The coexpression module of serum exosome metabolites is dysregulated in remote ischemic preconditioning (RIPC) participants. **A** Dendrogram of metabolite coexpression modules; **B** Log2 (fold change) distribution of metabolites in the blue, brown, green, red, turquoise, and yellow modules; **C** Pearson’s correlation coefficient between S0-S38 and module eigengene. S0: Group; S1: Visual selection reaction time (plain); S2: Blood oxygen saturation (SpO2) (4500 m); S3: Auditory simple reaction time (plain); S4: Nation; S5: Diastolic pressure (mmHg) (4500 m); S6: Blood oxygen saturation (SpO2) (plain); S7: Lake Louise Acute Mountain Sickness Scoring System (Gastrointestinal symptoms); S8: Digital decoding (4500 m); S9: Spatial memory (number of passes; 4500 m); S10: Digital decoding (plain); S11: Pulse (plain); S12: Systolic pressure (mmHg) (plain); S13: Target tracking (correct average dot; 4500 m); S14: Pulse (4500 m); S15: Auditory simple reaction time (4500 m); S16: Systolic pressure (mmHg) (4500 m); S17: Left prefrontal cerebral oxygen rSO2% (plain); S18: Weight; S19: Left prefrontal cerebral oxygen rSO2% (4500 m); S20: Height; S21: Diastolic pressure (mmHg) (plain); S22: Manual dexterity (dominant hand; 4500 m); S23: Right frontal cerebral oxygen saturation rSO2% (4500 m); S24: Target tracking (correct average dot; plain); S25: Target tracking (total average dot; plain); S26: Manual dexterity (non-dominant hand; 4500 m); S27: Spatial memory (number of passes; plain); S28: Spatial memory (plain); S29: Target tracking (total average dot; 4500 m); S30: Manual dexterity (non-dominant hand; plain); S31: Visual selection reaction time (4500 m); S32: Lake Louise Acute Mountain Sickness Scoring System (Dizziness); S33: Lake Louise Acute Mountain Sickness Scoring System (Headache); S34: Spatial memory (4500 m); S35: Right frontal cerebral oxygen saturation rSO2% (plain); S36: Lake Louise Acute Mountain Sickness Scoring System (Fatigue and/or weakness); S37: Manual dexterity (dominant hand; plain); S38: Lake Louise Acute Mountain Sickness Scoring System (Total scores)

To better understand the role of serum exosomal metabolite dysregulation in ischemic status, we used WGCNA to assign individual metabolites to coexpression modules, which were identified as 6 modules (Fig. 2A). The results suggested that two modules were significantly correlated with ischemic status: the modules represented in red and yellow showed decreased levels (Fig. 2B, Additional file 2). As shown in Fig. 2C, the red module had a significant association with auditory simple reaction time (plain); Nation; Lake Louise Acute Mountain Sickness Scoring System (Gastrointestinal symptoms); digital decoding (4500 m); diastolic pressure (mmHg) (plain); right frontal cerebral oxygen saturation rSO2% (4500 m); spatial memory (number of passes; plain); target tracking (total average dot; 4500 m); visual selection reaction time (4500 m); and Lake Louise Acute Mountain Sickness Scoring System (total scores). The yellow module had a significant association with systolic pressure (mmHg) (4500 m); manual dexterity (nondominant hand; plain); visual selection reaction time (4500 m); Lake Louise Acute Mountain Sickness Scoring System (fatigue and/or weakness); and manual dexterity (dominant hand; plain).

### Exosomal metabolites as biomarkers for RIPC

**Fig. 3:**
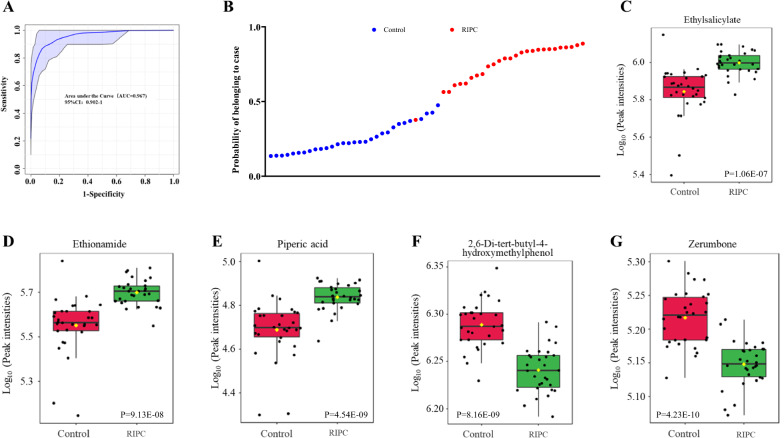
Serum exosomal metabolites as biomarkers to differentiate between remote ischemic preconditioning (RIPC) participants and controls. **A** ROC curves were utilized to evaluate the accuracy of the analysis of a cluster of 5 metabolites; **B** A scatter plot of the probability of participants belonging to cases by the 5 metabolites; Boxplot of **C** ethyl salicylate (*P* = 1.06E-07); **D** ethionamide (*P* = 9.13E-08); **E** piperic acid (*P* = 4.54E-09); **F** 2,6-di-tert-butyl-4-hydroxymethylphenol (*P* = 8.16E-09); **G** zerumbone levels (*P* = 4.23E-10). AUC, area under curve; ROC, receiver operating characteristic

We explored whether exosomal metabolites could serve as biomarkers to differentiate between control and RIPC participants. A total of 87 metabolites were analyzed for potential metabolite biomarkers, and 5 metabolites were selected as the optimal set of metabolites. We used the 5 metabolites to draw an ROC curve, and the area under the curve (AUC) was 0.967 (95% CI, 0.98–1.0) (Figs. 3A, B). The metabolites ethyl salicylate, ethionamide, piperic acid, 2,6-di-tert-butyl-4-hydroxymethylphenol, and zerumbone were identified as the optimal set of metabolites to distinguish the control and RIPC participants (Figs. 3C–G).

### Differential expression of serum exosomal proteins

**Fig. 4:**
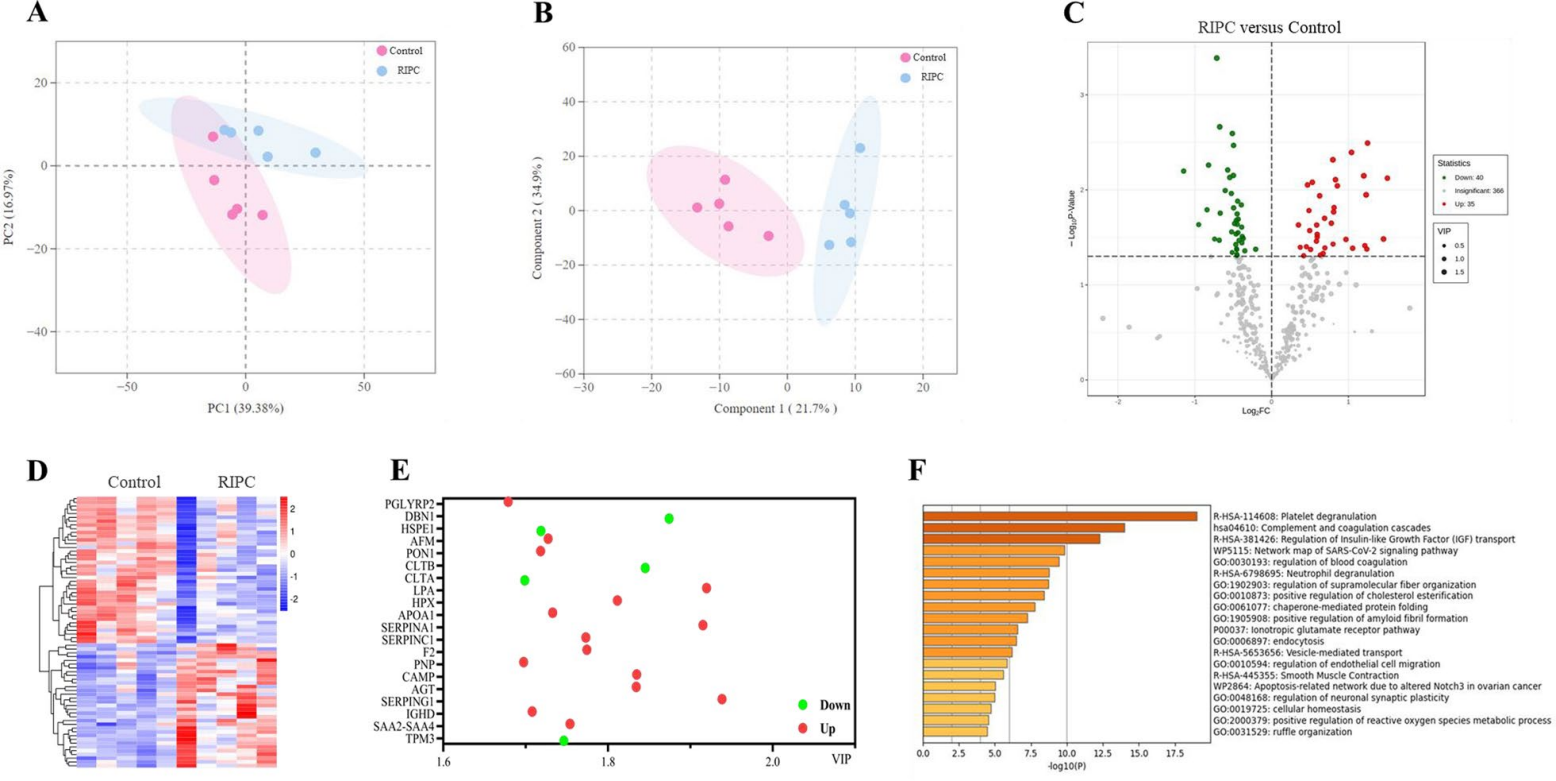
Bioinformatic screening for differential expression of proteins in serum exosomes. **A** Principal component analysis (PCA) and **B** orthogonal partial least squares-discriminant analysis (OPLS-DA) model plot based on the metabolites evaluated in the participant set; **C** volcano plot displaying differences in protein levels between RIPC participants and controls for the participant set; **D** heatmaps of the cluster analysis data; volcano plot displaying proteomics differences between RIPC participants and controls; Metascape enrichment network analysis for **E** top 20 enrichment metabolites and **F** top 20 enrichment pathways

We performed LC‒MS/MS to validate the serum exosomal proteomic profiles in RIPC participants. Plots of PCA scores showed a separation of proteome profiles for controls and preconditioning participants (Fig. 4A). An OPLS-DA model was performed to identify differentially expressed exosomal proteins (Fig. 4B). Of these, 40 were upregulated and 35 were downregulated in the RIPC participants compared to the controls (Figs. 4C, D). Then, we used Metascape enrichment analysis to assess the differentially expressed proteins. The top 20 enrichment proteins included peptidoglycan recognition protein 2 (PGLYRP2), drebrin 1 (DBN1), heat shock protein family E member 1 (HSPE1), afamin (AFM), and paraoxonase 1 (PON1). (Fig. 4E). The bioinformatics analyses showed the top 20 enrichment pathways, including platelet degranulation, complement, and coagulation cascades, regulation of insulin-like growth factor (IGF) transport, neutrophil degranulation, regulation of supramolecular fiber organization, endocytosis, and vesicle-mediated transport. (Fig. 4F).

### Integrative analysis of proteomics and metabolomics

**Fig. 5:**
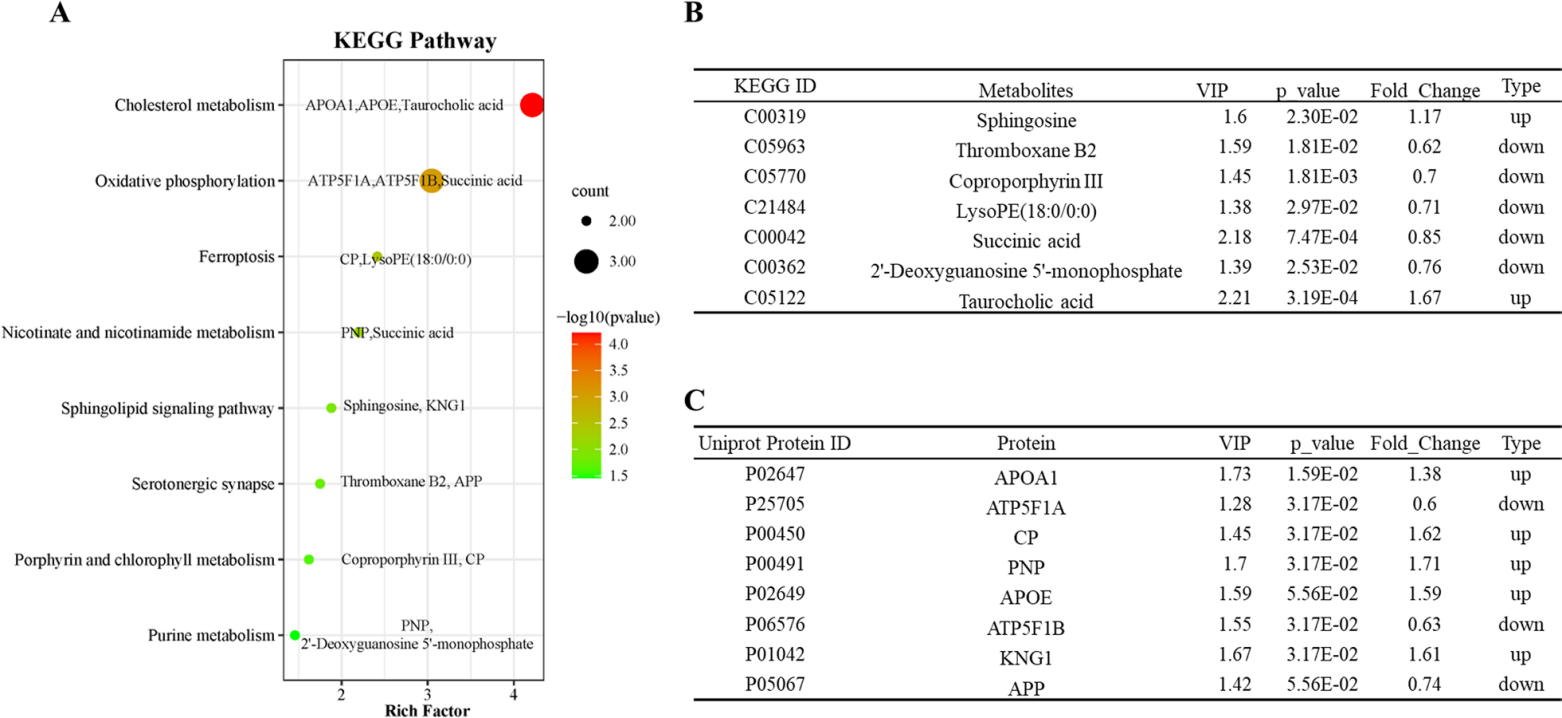
KEGG pathway enrichment analysis of multigroup analysis integrating metabolic and proteomic data. **A** Integrative analysis of proteomics and metabolomics; the coregulated enrichment of **B** metabolites and **C** proteins. The node size is proportional to enrichment pathways; the bubble color represents the *P* value, and the color change from green‒red represents a small to large transition of the -log10 (*P* value). KEGG, Kyoto Encyclopedia of Genes and Genomes

To establish a comprehensive profile of ischemia and identify the relationship between metabolites and proteins, a multigroup analysis integrating metabolic and proteomics data was conducted based on the same samples. KEGG pathway enrichment analysis showed that the coregulated features were mainly involved in cholesterol metabolism, oxidative phosphorylation, ferroptosis, nicotinate and nicotinamide metabolism, sphingolipid signaling pathway, serotonergic synapse, purine metabolism, etc. (Fig. 5A). Metabolites mostly included sphingosine, thromboxane B2, coproporphyrin, succinic acid, and taurocholic acid. (Fig. 5B). The coregulated proteins mainly included apolipoprotein A1 (APOA1), ceruloplasmin (CP), purine nucleoside phosphorylase (PNP), apolipoprotein E (APOE), and amyloid beta precursor protein (APP) (Fig. 5C, Additional file 2).

## Discussion

It is well known that RIPC protects the brain against ischemic injury [31]. Exosomes are extracellular vesicles released into the blood that transfer signals via cell communication [32]. In this study, we performed UPLC‒MS/MS and LC‒MS/MS to analyze the serum exosome metabolomic and proteomic profile associated with RIPC-mediated neuroprotection. The results showed differential metabolite and protein profiles in the serum exosomes under RIPC conditions. Briefly, 87 (56 with increased levels and 31 with decreased levels) differential metabolites were observed between RIPC participants controls. Regarding the proteomic results, 75 proteins (40 were upregulated and 35 were downregulated) showed differential expression between RIPC participants and controls. Further analysis suggested that the enriched pathways included tyrosine metabolism, sphingolipid metabolism, serotonergic synapse, pathways of multiple neurodegeneration diseases, Parkinson's disease, and GABAergic synapse. The proteomic functions included actin cytoskeleton organization, hemostasis, complement and coagulation cascades, vesicle-medicated transport, and wound healing. Integrative analysis of proteomic and metabolomic results showed that the coregulated features were mainly involved in oxidative phosphorylation, ferroptosis, nicotinate and nicotinamide metabolism, sphingolipid signaling pathway, serotonergic synapse, and purine metabolism. The bioinformatics analyses showed the top 20 enrichment pathways, including complement and coagulation cascades, regulation of IGF transport, neutrophil degranulation, endocytosis, and vesicle-mediated transport. Taken together, data from this study showed the dysregulation of serum exosomal metabolites and proteomic contents in RIPC.

RIPC caused by transient cerebral ischemia/reperfusion has a protective effect on brain injury induced by ischemic stroke [33]. Preconditioning leads to a protective phenotype labeled ischemic tolerance. The stimulation of RIPC induces tolerance by activating a large number of proteins, receptors, transcription factors, and other biological molecules and ultimately results in genome reprogramming [34]. Exosomes are involved in intercellular communication between local and distant cells [35]. Other forms of intercellular communication, including hormones, growth factors, cytokines, and direct interactions, play a critical role in how multicellular organisms can function as a single system [36]. They package active cargo such as proteins, nucleic acids, and lipids, deliver them to other neighboring or distant cells, and regulate the function of receptor cells through their delivery [37]. While this form of communication occurs between physiologically healthy cells, diseased cells package their active machinery in exosomes and transport them to other healthy cells to play a role in disease metastasis [38].

The pathophysiology of ischemic stroke is very complex, including early and late processes such as cell apoptosis, neuroinflammation, neurovascular repair, and regeneration [39]. Our results revealed that a series of metabolic pathways are closely related to cerebral ischemia/reperfusion injury. Cerebral ischemia/reperfusion injury involves the interaction between oxidative stress and inflammation, which is the basis of the development of the ischemic stroke cascade reaction [40]. In addition, ischemia/reperfusion injury induces a decrease in tryptophan and tyrosine levels, while the ability to synthesize serotonin decreases in the brain [41]. Moreover, sphingolipids are an important structural component of cell membranes, which plays an essential role in controlling the signal transduction of cell proliferation, differentiation, and apoptosis [42]. Moreover, there is a connection and/or cascade reaction among tyrosine metabolism, sphingolipid metabolism, serotonergic synapses, pathways of multiple neurodegeneration diseases, and GABAergic synapses [43, 44]. It is also applicable to our metabolism results from serum exosomes of RIPC participants. In this study, we found that RIPC may change the levels of a series of metabolites in serum exosomes to adapt to cerebral ischemia/reperfusion injury.

In addition to proteomic alterations, such as tyrosine phosphorylation, in the pathogenesis of ischemic stroke, growth factors or neurotrophic factors, including IGF, FGF, and BDNF, can reduce cell damage by inhibiting the tyrosine kinase receptor-activated apoptosis pathway [45]. IGF is a highly effective antiapoptotic factor in eukaryotic cells. It is considered to be a neuroprotective target in inflammatory and excitotoxic conditions. Therefore, IGF can reduce tissue and cell damage induced by ischemia and reperfusion [46]. In this study, our results demonstrate that the primary functions involved in RIPC included complement and coagulation cascades, regulation of IGF transport, neutrophil degranulation, endocytosis, and vesicle-mediated transport, which may participate in the potential role of RIPC. Our metabolomics and proteomics data showed that RIPC induces an ischemic cascade, and these peripheral signals are transmitted to the brain through exosomes to protect the brain against the effects of ischemia/reperfusion on the body. In addition, integrative analysis of proteomics and metabolomics showed that the differential metabolites and proteins connected to form a network under RIPC conditions. Our metabolomics data may provide a multitarget neurovascular unit protection strategy for ischemic stroke.

RIPC is an endogenous protective pathway of cerebral ischemia‒reperfusion injury [47]. The protective effect of RIPC on cerebral ischemia is mainly related to a variety of biological molecules and signaling pathways [48]. During ischemia, the tissues adapt to anaerobic metabolism [49]. The restoration of the blood supply causes the oxygen supply to exceed the requirements, which leads to the production of superoxide free radicals, causing oxidative stress. The key event involved in the initial stage of reperfusion injury is the activation of macrophages, which leads to endothelial injury and further release of proinflammatory cytokines [50, 51]. In this study, a differential expression profile of blood exosome-derived metabolites and proteins was observed under RIPC conditions. We found some differential metabolites and proteins, such as Theobromine, cyclo gly-pro, HPX, and ApoA1, that are associated with neuroprotective benefits in ischemia/reperfusion injury. It has been reported that Theobromine is a natural stimulant and vasoactive alkaloid that can prevent ischemic injury [52]; Cyclo gly-pro has a neuroprotective effect on hypoxic-ischemic brain injury in rats [53]; HPX is a rate-limiting enzyme that eliminates excessive free hemoglobin during ischemic stroke [54]; ApoA1 is the main transport protein for high-density lipoprotein macromolecules and significantly reduces the infarct volume and the transformation rate of hemorrhage by decreasing neutrophil recruitment [55]. RIPC may regulate the expression of these metabolites and/or proteins to induce ischemic tolerance to subsequent hypoxic injury.

Through the integrative analysis of blood exosomal metabolome and proteome data, 8 significantly perturbed pathways were identified. Among them, APOA1, APOE, and taurocholic acid were involved in cholesterol metabolism. Cholesterol metabolism was found to be significantly related to adverse outcomes of ischemic stroke [56]. In addition, ApoE is a multifunctional protein that plays a key role in cholesterol metabolism [57]; a higher level of APOA1 is considered to be protective against ischemic stroke [58], and taurocholic acid can lower postprandial lipemia [59]. Our results showed that ApoE, APOA1, and taurocholic acid showed higher levels in RIPC participants than in controls, which may have protective effects when participants are exposed to RIPC conditions. Furthermore, ATP synthase, H + transporting, mitochondrial F1 complex, alpha subunit 1 (ATP5A1; ATP5A1A and ATP5A1B) is positively correlated with the oxidative phosphorylation pathway in cells [60]; the reduction in succinic acid levels reduces oxidative phosphorylation [61]. Our results revealed that ATP5A1A, ATP5A1B, and succinic acid were less involved in the oxidative phosphorylation pathway, which is consistent with a previous study that reported that RIPC involves beneficial effects on oxidative phosphorylation of mitochondria [62]. Our results suggested that RIPC is involved in cholesterol metabolism and the oxidative metabolism pathway transmitted by blood exosomes. In addition, blood exosomes may play critical roles in the transfer of signals during the ischemia/reperfusion process.

RIPC refers to a brief episode of exposure to potential adverse stimulation and prevents injury during subsequent exposure. The protective mechanisms include stimulation of nitric oxide synthase, an increase in the levels of antioxidant enzymes, and downregulation of proinflammatory cytokines [2]. In this study, five potential metabolite biomarkers that separated RIPC from control individuals were identified. Our results showed that ethyl salicylate, ethionamide, and piperic acid levels were higher, and 2,6-di-tert-butyl-4-hydroxymethylphenol and zerumbone were lower under RIPC conditions. It has been reported that ethyl salicylate functions as an antibacterial and anti-inflammatory component for the treatment of tuberculous meningitis [63]. Furthermore, ethionamide has antibacterial and anti-inflammatory effects [64]. Piperic acid is indicated to have antinociceptive and anti-inflammatory activities [65]. These three metabolites may provide protective benefits when participants are exposed to RIPC conditions. Additionally, a limitation of this study is that the sample size was relatively small, which requires future large studies to verify the data from the present study.

The metabolomics and proteomics analysis of serum exosomes following RIPC has led to insight into metabolism during RIPC and the possible enrichment pathways of metabolites and proteins that are relevant to ischemia‒reperfusion damage. Our findings provide a better understanding of the pathophysiologic effects of RIPC and may facilitate the improvement of diagnostics and therapeutics of cerebral ischemia‒reperfusion injury for human clinical application. In addition, our data suggest that serum exosomal metabolites are promising biomarkers for RIPC and may provide a new treatment strategy for future cerebral ischemia‒reperfusion injury.

## Supplementary Information

**Additional file 1.** Exosome Characterization. (A) Serum exosome observation by electron microscopy and ×3000 magnification. (B) Serum exosome validation by nanoparticle tracking device—ZetaView. Note that the particles peak around at 100 nm

**Additional file 2.** Supplementary Table 1 Expression levels of exosomemetabolites in the two down regulated modules (red, yellow); Supplementary Table 2. Top 20 Metascape enrichment pathway for DE proteins; Supplementary Table 3. Top 20 KEGG pathway for DE metabolites; Supplementary Table 4. KEGG pathway for DE metabolite-gene interaction analysis

## Data Availability

Not applicable.

## Abbreviations

AFM: Afamin
APOA1: Apolipoprotein A1
APOE: Apolipoprotein E
APP: Amyloid beta precursor protein
ATP5A1: ATP synthase, H + transporting, mitochondrial F1 complex, alpha subunit 1
AUC: Area under curve
BH: Benjamini-Hochberg
CP: Ceruloplasmin
DBN1: Drebrin 1
GO: Gene Ontology
HSPE1: Heat shock protein family E member 1
IGF: Insulin-like Growth Factor
KEGG: Kyoto Encyclopedia of Genes and Genomes
LC–MS/MS: Liquid chromatography-tandem mass spectrometry
OPLS-DA: Orthogonal partial least squares discriminant analysis
PCA: Principal components analysis
PGLYRP2: Peptidoglycan Recognition Protein 2
PNP: Purine nucleoside phosphorylase
PON1: Paraoxonase 1
RIPC: Remote ischemic preconditioning
ROC: Receiver operating characteristic
UPLC-MS/MS: Ultraperformance liquid chromatography-tandem mass spectrometry
WGCNA: Weighted Gene Coexpression Network Analysis
