# Supplementary material for: Identification of serum exosomal metabolomic and proteomic profiles for remote ischemic preconditioning
Source: J Transl Med. 2023 Apr 3;21:241. doi: 10.1186/s12967-023-04070-1 (PMC10069038; doi:10.1186/s12967-023-04070-1)
Supplement: Supplementary file 1 — Additional file 1. Exosome Characterization. (A) Serum exosome observation by electron microscopy and ×3000 magnification. (B) Serum exosome validation by nanoparticle tracking device—ZetaView. Note that the particles peak around at 100 nm [file 12967_2023_4070_MOESM1_ESM.pdf]

Supplementary Figure

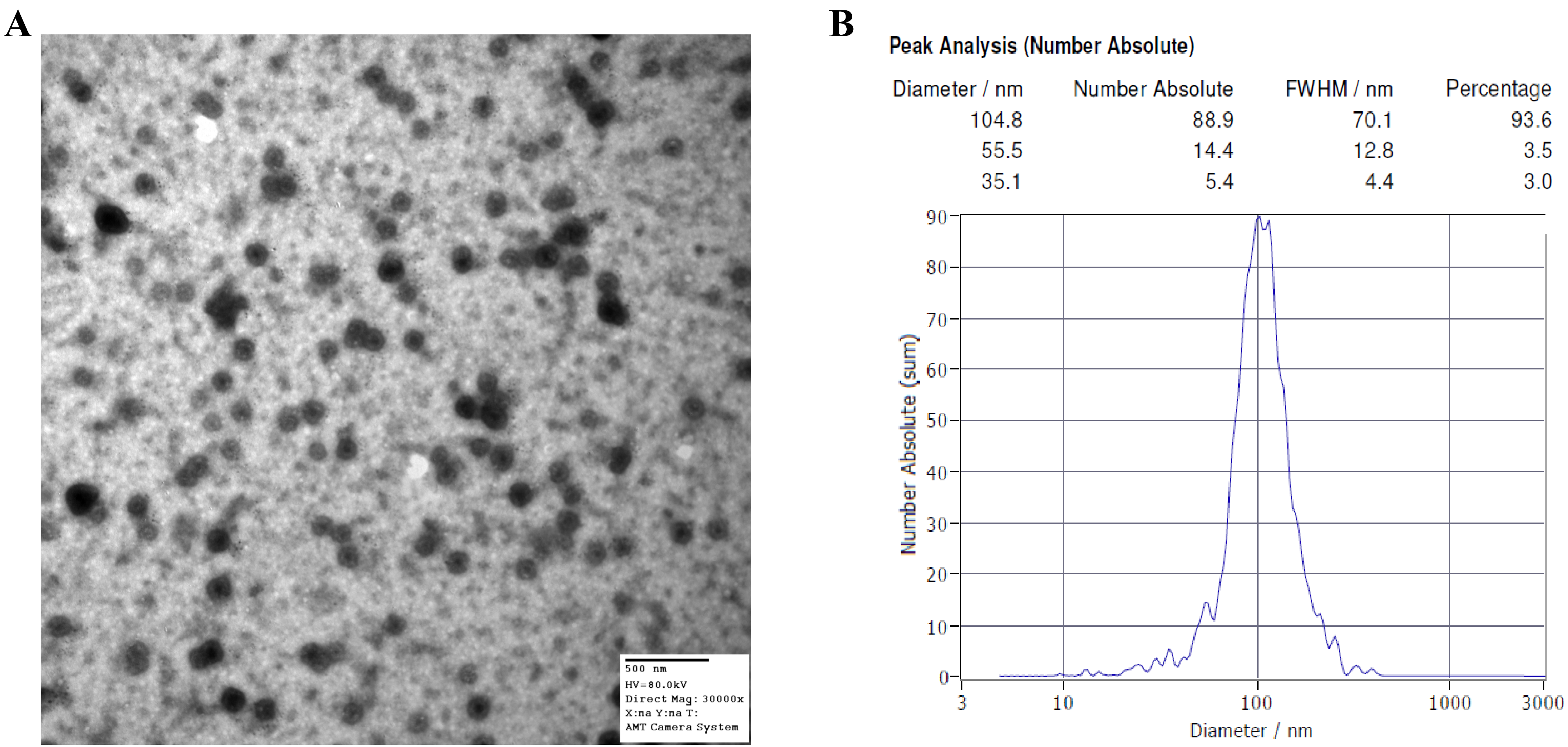


Exosome Characterization. (A) Serum exosome observation by electron microscopy and ×3000 magnification.
(B) Serum exosome validation by nanoparticle tracking device—ZetaView. Note that the particles peak around at 100 nm.
